# Empirical Bayes accomodation of batch-effects in microarray data using identical replicate reference samples: application to RNA expression profiling of blood from Duchenne muscular dystrophy patients

**DOI:** 10.1186/1471-2164-9-494

**Published:** 2008-10-20

**Authors:** Wynn L Walker, Isaac H Liao, Donald L Gilbert, Brenda Wong, Katherine S Pollard, Charles E McCulloch, Lisa Lit, Frank R Sharp

**Affiliations:** 1 Department of Neurology and MIND Institute, University of California at Davis, Sacramento, California, USA; 2 Genome Center and Department of Statistics, University of California, Davis, CA, USA; 3 Department of Pediatric Neurology, Cincinnati Children's Hospital and Medical Center, University of Cincinnati, Cincinnati, OH, USA; 4 Department of Epidemiology and Biostatistics, School of Medicine, University of California, San Francisco, CA, USA

## Abstract

**Background:**

Non-biological experimental error routinely occurs in microarray data collected in different batches. It is often impossible to compare groups of samples from independent experiments because batch effects confound true gene expression differences. Existing methods can correct for batch effects only when samples from all biological groups are represented in every batch.

**Results:**

In this report we describe a generalized empirical Bayes approach to correct for cross-experimental batch effects, allowing direct comparisons of gene expression between biological groups from independent experiments. The proposed experimental design uses identical reference samples in each batch in every experiment. These reference samples are from the same tissue as the experimental samples. This design with tissue matched reference samples allows a gene-by-gene correction to be performed using fewer arrays than currently available methods. We examine the effects of non-biological variation within a single experiment and between experiments.

**Conclusion:**

Batch correction has a significant impact on which genes are identified as differentially regulated. Using this method, gene expression in the blood of patients with Duchenne Muscular Dystrophy is shown to differ for hundreds of genes when compared to controls. The numbers of specific genes differ depending upon whether between experiment and/or between batch corrections are performed.

## Background

Non-biological experimental variation is commonly observed in microarray data processed in different batches. A batch is defined as a set of microarrays that are processed together within a single experiment. In this report we define an experiment as an individual study conducted at one site at one time. An experiment often has many samples processed in multiple batches. Different batches are processed at different times or by different operators. Batch effects are caused by many factors such as the methods for RNA isolation, amplification and target labeling, and array processing and scanning. Several methods have been proposed that can adjust for batch effects provided a large number of samples (> 25) are included in each batch [1,2]. More recently, an empirical Bayes method has been described [3] that adjusts for batch effects even when the number of samples in each batch is small (< 10).

**Figure 1:**
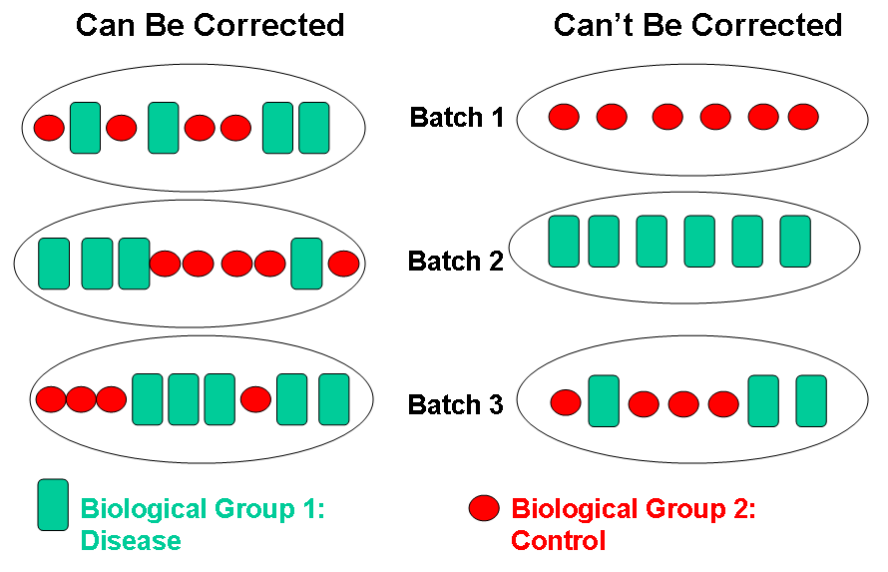
**Batch processing of microarray samples from different biological groups**. Examples of experimental designs that can be corrected for batch effects (left panel) and cannot be corrected for batch effects (right panel).

The aforementioned methods can adjust for batch effects provided that samples from each biological group are represented in every batch. The left panel of Figure 1 shows such an experimental design. All three batches contain samples from each of the two biological groups (disease and control). In contrast, the experiment in the right panel of Figure 1 is an example where it is not possible to distinguish differences in gene expression that are due to batch effects from those that are due to the underlying biology. Confounding batch effects are even more of a problem when comparing array data from experiments conducted in different laboratories. Hence, researchers need to generate a new set of control samples for each separate experiment. If it were possible to correct for cross-experimental batch effects, however, a single set of patient control samples could be compared to sets of disease samples from multiple independent experiments, saving significant resources.

**Figure 2:**
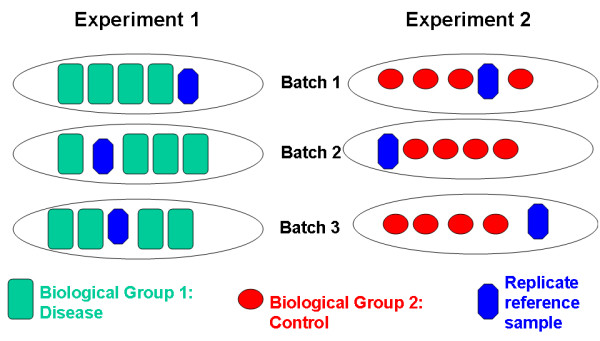
**Experimental design with reference samples**. This design enables the direct comparison of different biological groups drawn from independent experiments that would otherwise be incomparable.

In this report we present an experimental design that allows one to compare different biological groups drawn from independent experiments. The feature of our experimental design that allows samples to be compared even when control and experimental samples are in completely separate experiments is that identical replicate reference samples of pooled RNA are processed in each batch in every experiment. These reference samples are pooled RNA from the same tissue as are the disease and control experimental samples. In our study the experimental samples and reference samples are from human blood. Only a single reference sample is necessary to be included in each batch. Our experimental design is illustrated in Figure 2. We demonstrate models for estimating batch effects using the empirical Bayes approach of Johnson et al [3]. We focus on single-channel microarrays, though our methods could easily be extended to two-channel arrays.

## Results

Simulations and data analyses are used to study the properties of the proposed methods. These methods are an extension of the empirical Bayes batch effect adjustment proposed by Johnson et al [3].

### Empirical Bayes method adjusts gene expression in the proper direction

**Figure 3:**
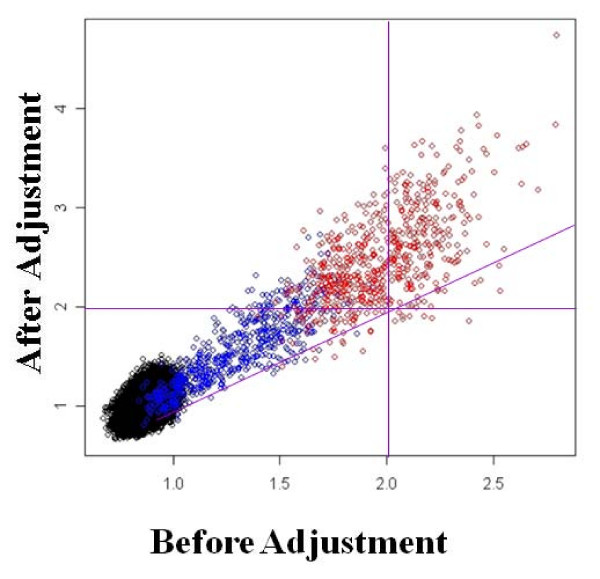
**Scatter plot of fold change values before and after batch adjustment for simulated data sets**. Genes are color coded according to their expected difference in expression level between patients and controls. Genes with the same expected level of expression in patients and controls are shown in black, while those with an expected 1.0 to 2.0 fold higher expression level in patients are in blue, and those with a 2.1 to 3.0 fold higher expression level are in red. This simulation is performed for data in which the batch effects artificially lowered the fold change values.

**Figure 4:**
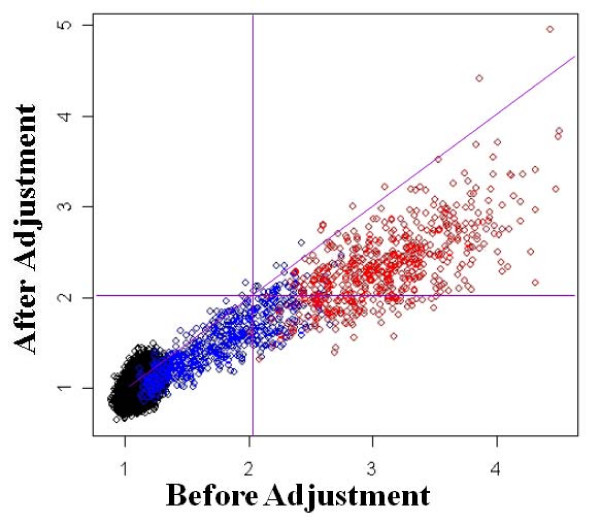
**Scatter plot of fold change values before and after batch adjustment for simulated data sets**. Genes are color coded according to their expected difference in expression level between patients and controls. Genes with the same expected level of expression in patients and controls are shown in black, while those with an expected 1.0 to 2.0 fold higher expression level in patients are in blue, and those with a 2.1 to 3.0 fold higher expression level are in red. This simulation is performed for data in which the batch effects artificially increased the fold change values.

We first show that the empirical Bayes method can correctly adjust for batch effects using two simulated data sets (Figures 3 and 4). For the first simulation, we select batch effect parameter values that lower the expression levels of patients relative to controls (Figure 3). We show that batch adjustment restores the fold change, leading to better power to detect truly differentially expressed genes. In the second simulation, we study the opposite scenario where batch effects elevate expression levels of patients relative to controls (Figure 4). Here, batch effects lead to false positives in the unadjusted data. Batch adjustment correctly (and conservatively) lowers the fold change values. In both cases, batch adjustment leads to more accurate conclusions.

For the first simulation, analysis of unadjusted data identifies only 265 (44%) of truly differential expressed genes as being differentially expressed with FDR adjusted p-value ≤ 0.05 and fold change > 2.0. Because the batch effects lowered the fold change values, all of the remaining simulated genes had fold change values of less than 2.0 (Figure 3). However, after batch adjustment, the vast majority of truly differentially expressed genes (528 out of 600) were identified as differentially expressed, with only 70 (0.1%) false positives. The ability of the method to correctly adjust for batch effects is further illustrated in Figure 3. This scatter plot of fold change values before and after batch adjustment shows that the majority of simulated expressed genes (all but 40 out of the 600 red points) are above the diagonal line, indicating that the batch adjustment method is adjusting the gene expression values in the proper direction. The red points in the upper left section of the plot separated by the two vertical purple lines correspond to the simulated expressed genes that were recovered as differentially expressed after batch adjustment.

In the second simulation, batch effects amplify the expression level differences between the two groups (Figure 4). Not surprisingly, all 600 simulated differentially expressed genes were declared as differentially expressed in the unadjusted data set. An additional 189 of the remaining genes were also identified as differentially expressed. After batch adjustment, 472 out of the 600 true positives were correctly identified, with only 45 false positives. This indicates that while the empirical Bayes method may be a bit stringent, it is very effective at removing false positives. The ability of the method to correctly adjust for batch effects is shown in Figure 4. Fold change values are lower in the adjusted data set relative to the unadjusted data set for all but seven of the 600 expressed genes, again indicating that the method adjusts the fold changes in the correct direction. The blue points in the lower right section of the plot separated by the two vertical purple lines are gene expression values for the simulated unexpressed genes that were correctly removed by the batch adjustment method (Figure 4).

### Empirical Bayes method removes cross-experiment batch effects

We next demonstrate on our experimental data set that the empirical Bayes method removes batch effects that occur between experiments. In this data there are two experiments, one that includes microarray data for four batches of control samples processed at the University of Cincinnati and a second that includes microarray data for patients with muscular dystrophy processed in five batches at UC Davis. We illustrate the successful removal of batch effects by comparing expression of differentially expressed genes before and after adjustment for batch effects.

**Figure 5:**
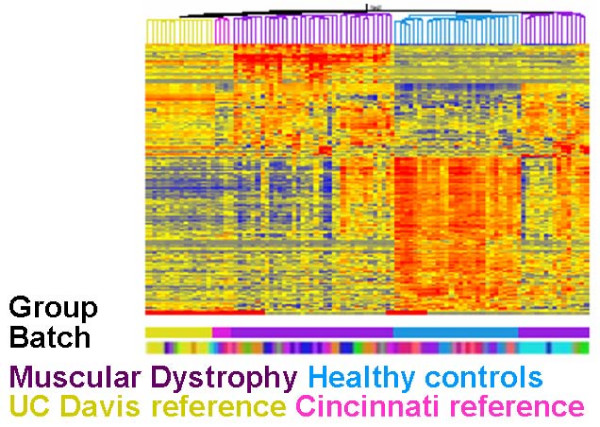
**Heat map of gene expression values for differentially expressed genes in muscular dystrophy data set before adjustment **for both within- and between-experiment batch effects. Two fold or greater increases of gene expression are shown in RED, and two fold or greater decreases of gene expression are shown in BLUE within the clusters. Note that the UC Davis reference sample group (yellow) completely separates from the Cincinnati reference sample group (pink).

For the unadjusted GCRMA-summarized expression values, a total of 527 genes are differentially expressed in patients compared to controls. Using the expression values for these genes only, we applied agglomerative hierarchical clustering with Pearson correlation to the 98 samples (Figure 5). The color-coded dendrogram and the upper color bar beneath the heat map show a clear separation of the muscular dystrophy patients (purple) from healthy control patients (light blue). However, the UC Davis reference arrays (gold) and the Cincinnati reference arrays (pink) also form separate groups. This is indicative that for this gene list there is a substantial amount of gene expression due to cross-experimental non-biological artifacts. Identical reference samples otherwise should have similar expression values and so should instead be randomly intermixed.

**Figure 6:**
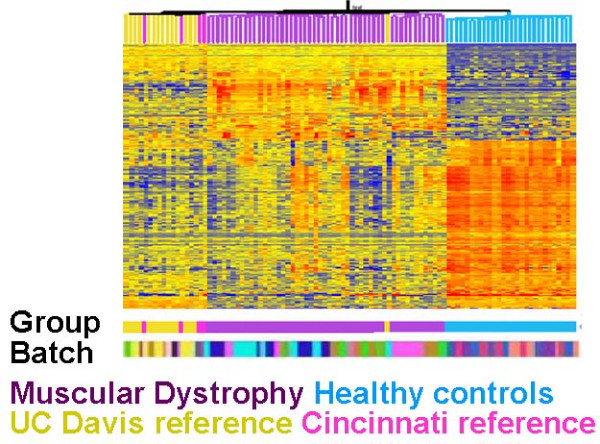
**Heat map of gene expression values for differentially expressed genes in muscular dystrophy data set after adjustment **for both within- and between-experiment batch effects. Two fold or greater increases of gene expression are shown in RED, and two fold or greater decreases of gene expression are shown in BLUE within the clusters. Note that the UC Davis reference sample group (yellow) is interspersed with the Cincinnati reference sample group (pink).

Using the empirical Bayes method to correct for both within and between experiment batch effects (Model 1), we identified 629 differentially expressed genes. Figure 6 is a clustered heat map based on these 629 genes. In the corresponding dendrogram the reference arrays (UC Davis is gold and Cincinnati reference samples are pink) are mixed, suggesting that the cross-experimental batch effects have been removed. The numbers of differentially expressed genes between the sets of reference samples also points to the successful removal of cross-experimental effects. While 85 genes were differentially expressed between the two groups of reference samples before the data was adjusted, there were no genes differentially expressed between UC Davis and Cincinnati reference samples after cross-experiment batch adjustment.

### Empirical Bayes method removes within-experiment batch effects

In this section we show that the empirical Bayes method successfully adjusts for within-experiment batch effects and that these can be of even greater magnitude than the between-experiment batch effects.

Table 1 shows the number of genes with significant within-experiment batch effects for the unadjusted and adjusted data sets. The numbers of genes for which the average gene expression in at least one batch is significantly different from the other batches (based on a one-way ANOVA with FDR followed by a fold change filter of 2.0) are given for each experiment. For the unadjusted data, 527 out of 54,675 genes were differentially expressed in at least one of the five batches processed at UC Davis, while 48 genes were for at least one of the four batches processed at Cincinnati. Interestingly, only three genes are in common between the two sets. Table 1 shows that there are comparable numbers of differentially expressed genes in at least one batch after adjusting only for between-experiment batch effects (Model 2). Comparing these large numbers of genes with significant gene expression in at least one batch to the number of genes differentially expressed between sets of replicate samples for the two experiments (85) suggests that both within- and between-experiment batch effects are of a sizeable magnitude in this data set.

**Table 1:** Numbers of differentially expressed genes between batches within a single experiment identified using ANOVA

**Adjustment Method**	**Cincinnati**	**UC Davis**	**Common**
None	48	527	3
t-test filter	0 (of 273)	42 (of 273)	0
EB: between experiment only	96	545	8
EB: within experiment only	0	4	0
EB: within and between experiment	0	0	0

Numbers are out of 54,675 probe sets unless otherwise specified. EB = empirical Bayes.

There is one batch processed at UC Davis that clearly has the most pronounced effects. Out of the 527 genes that were differentially expressed in at least one batch for the unadjusted data set, nearly all (517) were differentially expressed in the same batch. For the remaining four batches, the numbers of differentially expressed genes were many fewer – ranging between 27 and 112 for the four batches processed at UC Davis and between 4 and 28 for those processed at Cincinnati. The lower color bar beneath the heat map in Figure 6 (color-coded according to batch) also points to the pronounced effects in this one batch. The samples are interspersed for all batches except this one (colored teal). These results clearly point to the need for a method that is capable of removing both within and between experimental batch effects.

The numbers of differentially expressed genes are shown in Table 1 for the data sets adjusted for both types of batch effects (Model 1) and for within-experiment batch effects only (Model 3). For the data set adjusted only for within-experiment batch effects, there are only four genes that were differentially expressed in at least one batch for the UC Davis data set and no genes for the Cincinnati data set. These numbers show that the empirical Bayes method is very effective at removing within-experiment batch effects. When the data was adjusted for both types of batch effects, there were no differentially expressed genes across batches. Similarly, no genes were differentially expressed between UC Davis and Cincinnati reference samples after batch adjustment with Model 1.

### Comparison of the empirical Bayes method to the t-test filter

In this section we illustrate the advantages that the empirical Bayes method offers by comparing it to a more naïve approach which uses a t-test to filter out genes with significant differences in expression between reference arrays in the two experiments. Applying the t-test to the 527 genes identified as differentially expressed between patients and controls in the unadjusted data results in the removal of 254 genes with significant between-experiment batch effects. Out of the remaining 273 genes, while none show differential expression in at least one of the four batches processed in Cincinnati, 42 show differential gene expression in at least one of the five batches processed at UC Davis (Table 1). This is not surprising because the t-test removes genes with between-experiment batch effects but does not adjust for batch effects within an experiment. Moreover, the t-test filter has the additional limitation that it does not recover false negatives. The empirical Bayes approach identifies many more genes because it adjusts the values in the gene expression matrix instead of simply removing genes from a list.

### Adjusting for non-biological variation significantly alters lists of differentially expressed genes

**Figure 7:**
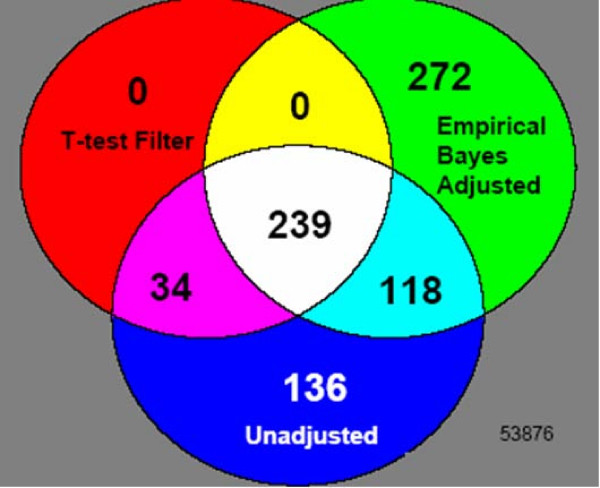
**Common Genes in lists of differentially expressed genes for three sets of gene expression values**: (1) unadjusted, (2) t-test Filtered, and (3) Empirical Bayes adjusted data. There are 239 genes common to all three gene lists.

Figure 7 shows the genes identified as differentially expressed in patients versus controls for the different methods explored in this paper: empirical Bayes adjustment for within and between experiment variation (Model 1: 629 genes), t-test filtering (273 genes), and unadjusted data (527 genes). The relatively small number common to all three gene lists (239 genes) illustrates the substantial effect of correcting for batch effects. Nearly 90% (239 out of 273) of the genes identified by the t-test filter were also identified by the empirical Bayes method, which identifies a number of additional genes.

**Figure 8:**
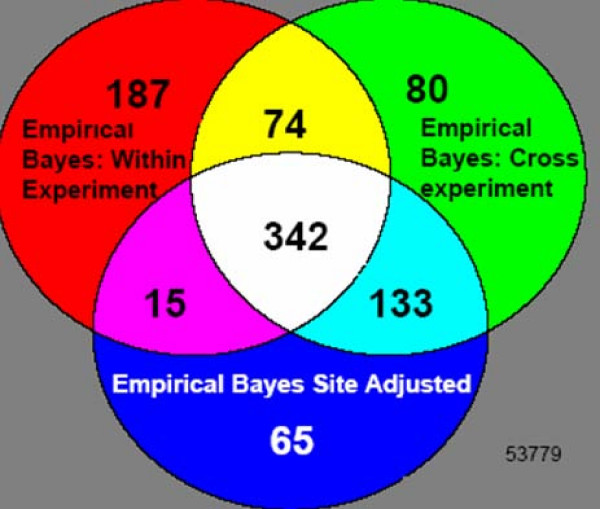
**Common Genes in lists of differentially expressed genes for three different empirical Bayes adjusted data sets**: (1) within experiment batch effects only, (2) cross-experiment site effects only, and (3) both cross-experiment site effects and within experiment batch effects. There are 342 genes common to all three gene lists.

Figure 8 shows the genes identified as differentially expressed in patients versus controls for the three different empirical Bayes adjustments: Model 1 (629 genes), Model 2 (555 genes), and Model 3 (618 genes). There are 342 genes common to all three gene lists. Out of these three methods, many more unique genes are identified when correcting only for within experiment batch effects (187) than there are for either of the methods that correct for cross-experiment variation (65 and 80 unique genes). Since there appear to be substantial cross-experiment batch effects in this data, it is likely that this list of 187 genes contains some false positives. Overall, our simulations suggest that the gene lists for empirical Bayes adjusted data contain fewer false positives and false negatives than the unadjusted analyses.

## Discussion and conclusion

In this report we have shown that with an appropriate experimental design and statistical methods it is possible to adjust for both within and between experiment batch effects and hence compare gene expression values between biological groups drawn from independent experiments. The unique feature of our experimental design that enables us to compare data from separate experiments is that identical replicate reference samples of pooled RNA (that are derived from the same tissue as are the experimental samples) are included in each batch within each experiment. The inclusion of the identical reference samples from the same tissue in every batch in every experiment allows us to adjust for non-biological variation and hence be able to distinguish differences in gene expression due to the underlying disease biology from those due to confounding batch effects. This approach enables us to compare the gene expression of samples from the two groups that would otherwise be incomparable due to confounding batch effects. The importance and uniqueness of this method are best understood when viewed in the context of the other methods described below.

Several computational methods have been developed to correct for non-biological variation. Alter et al. [1] used Singular Value Decomposition (SVD) to adjust for non-biological variation in yeast cell cycle data while others [4] applied this same method to correct for batch effects in a tumor data set. Alter et al. were able to adjust for non-biological variation by inferring that the combinations of genes and arrays that contributed the most to the variance correspond to non-biological artifacts. By filtering out these combinations of genes and arrays they were able to normalize the data and compare gene expression across arrays from different experiments. Because SVD finds the directions of greatest variation, this approach succeeded because in this case, the non-biological variation was the greatest source of variation. However, if the amount of biological variation happened to be greater than the non-biological variation, this approach would have likely failed to adjust non-biological variation. This motivated Benito et al. to apply a different approach, Distance Weighted Discrimination (DWD), to correct for systematic batch effects. In DWD, instead of adjusting the data based on the direction of maximal variation (as is done in SVD), data is adjusted according to the direction that maximizes the separation between two batches. While DWD can be applied to only two batches at a time, Benito et al. propose adjusting for three or more batches in a stepwise manner, adjusting the two most similar first, and then next most similar against the previously adjusted batches. It is unclear how successful their method is in removing batch effects when there are more than three batches.

The Bayesian approach developed by Johnson et al [3] is based on a location and scale (L/S) model that allows a different mean and variance for each gene and batch (see [5] for review). It offers the advantage of circumventing the requirements for large sample sizes while providing robust batch adjustments for each gene by pooling information across genes in each batch when estimating the model parameters. Given our limited sample size in each batch as well as the limited number of replicate identical reference samples in each batch, this Bayesian approach is an appropriate choice. Since many experimental designs share these features, we expect the approach could be widely applicable. To fully validate the current methods, it will be important to compare the Bayesian approach developed by Johnson et al {3} to the current approach on a data set where both approaches could be used.

This Bayesian approach is particularly advantageous for the analysis of our data set which had a particularly limited number of reference samples – the minimum number of one reference sample per batch. Because in the first step of the parameter estimation procedure, the pooled variance for each gene is calculated across *all *samples, the multiplicative batch effect (i.e. the variance) can still be estimated even when there is only a single reference sample in each batch. However, an experimental design would ideally include more than one reference sample per batch as a preventative measure in case one of the reference samples was of poor quality. In this study we are able to justify the use of a single reference sample based on the results of an ANOVA analysis of the batch adjusted data. The bottom row of Table 1 (which shows the numbers of differentially expressed genes between batches within a single experiment using ANOVA) shows that after batch effects have been adjusted for, there are no differentially expressed genes between batches for the disease samples, or between batches for the healthy control samples. This suggests that if the reference sample itself deviated from the other reference samples, the disease samples in this batch also deviated from the other disease samples in the other batch in a similar manner. By adjusting using the reference sample, the biases were correctly compensated. In subsequent studies we are addressing the optimal number of reference samples to include in each batch.

A related concern is that the estimate of the variance for many genes might be unusually large relative to the differences in the batch means because there was only one reference sample used. In this scenario the adjustment method would often times only add noise and reduce sensitivity. It is important to note that for the observed estimated parameter values in our data set, it is infrequent for the batch variances to be large relative to the differences in the batch means. Again, even though there is only one reference sample per batch, the variance estimate is likely stabilized by the pooling of samples.

The methods described above have been applied in the context of adjusting batch effects within a single experiment. Existing methods for comparing microarray data across independent experiments include meta-analysis where sets of genes that are consistently differentially expressed across multiple experiments are identified. In this approach, statistical measures of differential expression generated from data sets derived from independent experiments are compared instead of the gene expression data matrices from different data sets. Rhodes et al. [6] developed a statistical approach to compare multiple independent microarray data sets and applied it to identify sets of consistently differentially expressed genes across four independent prostate cancer gene data sets. In a subsequent study [7], they applied a similar method to identify cancer "meta-signatures" – sets of genes that are enriched in gene lists from many cancer studies.

The meta-analysis approaches described above have been very useful in comparing independent microarray data sets-especially when the data sets were generated across different platforms. However, in these methods the gene expression data matrices for test samples from one experiment are not compared to the control samples from another experiment. In contrast, our experimental design enables such comparisons to be made between experiments using the same microarray platform and hence offers an enormous increase in the number of different gene expression comparisons that can be made between data sets drawn from independent microarray studies. This makes it possible to test many more hypotheses for differences in gene expression. For example, suppose two independent studies were performed, each comparing a different disease group to healthy controls. If identical reference samples were included in each of these experiments, it would be possible to directly compare gene expression values between the two disease groups and identify genomic signatures that distinguish one disease group from the other in addition to the genomic signatures that distinguish each disease group from the healthy individuals.

The proposed experimental design has substantial impact for the use of microarrays in clinical studies. Because it is possible to compare gene expression values for disease and control samples drawn from separate experiments, it is possible to recycle expression data for one set of controls, comparing it to disease gene expression data sets for follow-up experiments. This would eliminate the burden and expense of reprocessing control samples each time an experiment is repeated. Furthermore with the ability to re-use a single control gene expression data set, it becomes possible to define a single standard control population for a disease study and establish a universal reference data set of gene expression values. This would dramatically increase the utility of microarrays in clinical diagnostics.

## Methods

### Microarray Data

Gene expression was measured using Affymetrix U133plus2 microarrays on 98 human blood samples in two separate experiments. This research in humans was carried out in compliance with the Helsinki Declaration. In one experiment, 66 arrays were processed at the University of California at Davis (UC Davis) in six batches. Fifty-one arrays probed independent samples from teenagers with Duchenne Muscular Dystrophy ('patients'). The other 15 arrays probed an identical reference sample generated at UC Davis which consists of pooled RNA isolated from four adults ('reference'). In five of the batches processed at UC Davis one reference array was included, and the sixth batch is composed entirely of reference arrays. In a separate experiment conducted at the University of Cincinnati, there were 32 arrays processed in four separate batches with each batch including between seven and nine arrays. 28 out of the 32 arrays probed samples from healthy teenagers ('controls'). Four arrays (one per batch) probed the same reference sample used at UC Davis.

Sample processing and array hybridization were performed according to standard Affymetrix protocols. Probe level data were summarized into a single expression value for each gene on each array using GCRMA in GENESPRING 7 (Agilent Technologies, ). Pre-processing involved non-linear background reduction utilizing probe DNA sequences, quantile normalization, and summarization by median polishing [8,9]. All of the primary expression data are deposited in the Gene Expression Omnibus (GEO) database or can be obtained from the authors upon request.

### Empirical Bayes Method for Batch Adjustment

Our goal is to identify genes that are differentially expressed in patients relative to controls.

Model Formulation: The mathematical model used in the empirical Bayes method [3] is

(1) *Y*_*ijg *_= *α*_*g *_+ *Xβ*_*g *_+ *γ*_*ig *_+ *δ*_*ig*_*ε*_*ijg*_

where *Y*_*ijg *_is the expression value for gene *g *for sample *j *from batch *i*, *α*_*g *_is the overall mean, *X *is a design matrix, and *β*_*g *_is the vector of regression coefficients corresponding to *X*. *γ*_*ig *_measures the additive batch effect of batch *i *for gene *g *which represents the location (mean) of the adjustment for this batch and is assumed to follow a normal distribution. *δ*_*ig *_measures the multiplicative batch effect, which is assumed to follow an inverse gamma distribution. The error terms, *ε*_*ijg*_, are assumed to follow a normal distribution with expected value of zero and variance *σ*_*g*_^2^.

We employ three versions of the above model to derive three different sets of empirical Bayes adjusted data: one adjusted for both within and between experiment non-biological variation, a second to adjust for only the variation that occurs between experiments, and a third to adjust only for the within experiment batch effects. We evaluate these three different formulations in order to assess and compare the relative effects of adjusting for the within and between experiment non-biological variation.

Model 1: within and between experiment effects: In Equation 1 gene *g *has parameters *γ*_*ig *_and *δ*_*ig *_for each of the ten distinct batches. There are a total of three regression coefficients in the vector of parameters for the biological group covariates, *β*_*g*_, one that specifies whether a sample is the reference, and two that specify disease state (healthy, Duchenne, or non-Duchenne muscular atrophy).

Model 2: between experiment effects only: The model formulation is the same as above except that there are only two values for *γ*_*ig *_and *δ*_*ig *_per gene, one for each experiment. In this model, each experiment is considered as one batch and batch effects within each experiment are not modeled.

Model 3: within experiment effects only: For the case where the data is not adjusted for between-experiment batch effects, the reference sample is not used and so there are only two coefficients in the vector *β*_*g*_.

### Parameter Estimation

The model parameters are estimated as detailed by Johnson et al [3]. This process can be summarized in three steps. First, the genes are standardized so that they all have similar mean and variance. This step reduced the effects of gene-to-gene variation (due to differences in mRNA expression levels and probe sensitivities) that would otherwise bias the batch effect estimates. Standardizing involves first estimating the model parameters *α*_*g*_, *β*_*g*_, and *γ*_*ig *_for each gene by least squares constraining $\sum_{i}n_{i}{\hat{\gamma }}_{ig}=0$ The pooled variance for each gene across all samples is

(2) $${\hat{\sigma }}_{g}^{2}=\frac{1}{N}{\sum_{ij}\left(Y_{ijg}-{\hat{\alpha }}_{g}-X{\hat{\beta }}_{g}-{\hat{\gamma }}_{ig}\right)}^{2}$$

where *N *is the total number of samples. Then, the standardized data is

(3) $$Z_{ijg}=\frac{Y_{ijg}-{\hat{\alpha }}_{g}-X{\hat{\beta }}_{g}}{{\hat{\sigma }}_{g}}.$$

In the second step, for each gene, the batch *i *sample mean is estimated as

(4) $${\hat{\gamma }}_{ig}=\frac{1}{n_{i}}\sum_{j}Z_{ijg}$$

and the batch *i *sample variance as

(5) $${\hat{\delta }}_{ig}^{2}=\frac{1}{n_{i}-1}\sum_{j}(Z_{ijg}-{\hat{\gamma }}_{ig}{)}^{2}.$$

These estimates are used to estimate the parameters of the distributions of *γ*_*ig *_and *δ*_*ig *_using the method of moments. Then, using the parameter estimates for these two prior distributions along with Bayes theorem, posterior distributions for each *γ*_*ig *_and *δ*_*ig *_are calculated. Final estimates for the batch effect parameters, γ*_ig _and δ*_ig_, are estimated as the expected values of the posterior distributions. This empirical Bayes estimation procedure allows information from all genes to be used to estimate batch effects for each gene, providing more stable estimates than the standard sample mean and sample variance.

In the third step, the data is adjusted to remove batch effects. The empirical Bayes [3] batch adjusted data is given by

(6) $$Y_{ijg}^{*}=\frac{{\hat{\sigma }}_{g}}{{\hat{\delta }}_{ig}^{*}}(Z_{ijg}-{\hat{\gamma }}_{ig}^{*})+{\hat{\alpha }}_{g}+X{\hat{\beta }}_{g}.$$

We performed the above calculations using the COMBAT software developed by Johnson et al and written in R . This software features diagnostic plots that allow one to check the validity of the model assumptions as well as an alternate nonparametric Bayesian method (in case the assumptions for parametric formulation are invalid).

### Identifying Differentially Expressed Genes

We performed unpaired two-sample t-tests in GENESPRING 7 to find genes with different mean expression between disease groups. First, subjects with muscular dystrophy were compared to the control subjects using unadjusted GCRMA-summarized gene expression data. The same t-test was repeated using the various empirical Bayes batch adjusted gene expression values. P-values were corrected for mulitple comparisons with the Benjamini-Hochberg False Discovery Rate (FDR) technique. An FDR corrected p-value ≤ 0.05 was considered significant, which means that no more than 5% of the declared different genes are expected to represent false positives. We further filtered each of the above lists removing all genes with average fold change < 2.0.

Differentially expressed genes between batches within each experiment were identified using one-way ANOVA within GENESPRING with an FDR corrected p-value threshold of 0.05.

### Naïve t-test Batch Adjustment

We compare the proposed empirical Bayes approach to a simple method that filters out genes with significant cross-experimental batch effects based on a t-test comparing the 15 reference arrays from UC Davis to the four reference arrays processed at the University of Cincinnati. This t-test filter was applied to each gene that was differentially expressed between disease groups in the GCRMA-summarized unadjusted gene expression data set. Each gene that was found to be significantly different between experiments (unadjusted p-value ≥ 0.2) was removed from the gene list because of the ambiguity regarding the reason for its differential expression (underlying biology versus non-biological site-specific variation). The loose p-value threshold of 0.2 for removing genes was chosen because of the limited accuracy of the t-test with one of the sample sizes only being four.

### Simulations

We performed two simulations to assess the ability of the empirical Bayes method to accurately identify differentially expressed genes in the presence of confounding batch effects. In one simulation we tested the ability of the method to recover false negatives – genes whose differential expression is reduced due to batch effects. For the second simulated data set, we tested the ability of the method to remove false positives – genes whose differential expression is elevated due to batch effects.

For each simulated data set, we generated gene expression values for 54,675 probe sets for 28 controls, 51 patients, and 19 identical reference samples. Model parameter values were selected to reflect trends observed in the muscular dystrophy data set. Specifically, we selected parameter values for *α*_*g *_and *β*_*g *_to generate a set of genes with mean fold changes between the disease and healthy samples that range between 1.1 and 3.0 in increments of 0.1. We simulated 60 genes with each fold change to generate (1) 600 genes with fold changes greater than 2.0 (to reflect the approximate number of differentially expressed genes in the real data), and (2) 600 additional genes with fold changes below 2.0, the threshold we selected for differentially expressed genes. Parameter values for *α*_*g *_and *β*_*g *_for the remaining 53,475 genes were selected so that there are no differences in mean expression between the two groups. The errors, *ε*_*ijg*_, for each gene were randomly simulated from a normal distribution with mean 0 and standard deviation *σ*_*g*_, chosen to reflect the average standard deviation observed in the real data. Batch scale parameters, *δ*_*ig*_, were also selected to reflect a typical range of observed values.

For the first simulation, we selected *γ*_*ig *_values whose mean for the patients is lower than that for the controls by an amount that counter balances the average difference in mean expression between patients and controls for truly differentially expressed genes. For the second simulation we selected *γ*_*ig *_values so that the mean for the patients is higher than that for the controls by an amount that generates a difference in expression between patients and controls for truly non-differentially expressed genes equivalent to that seen in truly differentially expressed genes. In each simulation, we identify genes declared to be differentially expressed before and after batch adjustment and compare the numbers of genes that are correctly and incorrectly classified as being differentially expressed in each case.

## Abbreviations

FDR: False Discovery rate; RNA: ribonucleic acid; GCRMA: GC Robust Multiarray Average; ANOVA: analysis of variance.

## Authors' contributions

WLW and FRS were responsible for designing the study and devising the batch correction approach presented here. KSP and CEM made suggestions about the modeling and comparison of the Bayes and t-test approaches. BW and DLG recruited the control and Duchenne Muscular Dystrophy patients that were used for the application of the methods developed here. IL and LL aided in writing the manuscript and evaluating the proposed methods. All of the authors read the manuscript and made suggestions for improvements.
